# The Dynamics of Human Body Weight Change

**DOI:** 10.1371/journal.pcbi.1000045

**Published:** 2008-03-28

**Authors:** Carson C. Chow, Kevin D. Hall

**Affiliations:** Laboratory of Biological Modeling, National Institute of Diabetes and Digestive and Kidney Diseases, National Institutes of Health, Bethesda, Maryland, United States of America; University of California San Diego, United States of America

## Abstract

An imbalance between energy intake and energy expenditure will lead to a change in body weight (mass) and body composition (fat and lean masses). A quantitative understanding of the processes involved, which currently remains lacking, will be useful in determining the etiology and treatment of obesity and other conditions resulting from prolonged energy imbalance. Here, we show that a mathematical model of the macronutrient flux balances can capture the long-term dynamics of human weight change; all previous models are special cases of this model. We show that the generic dynamic behavior of body composition for a clamped diet can be divided into two classes. In the first class, the body composition and mass are determined uniquely. In the second class, the body composition can exist at an infinite number of possible states. Surprisingly, perturbations of dietary energy intake or energy expenditure can give identical responses in both model classes, and existing data are insufficient to distinguish between these two possibilities. Nevertheless, this distinction has important implications for the efficacy of clinical interventions that alter body composition and mass.

## Author Summary

Understanding the dynamics of human body weight change has important consequences for conditions such as obesity, starvation, and wasting syndromes. Changes of body weight are known to result from imbalances between the energy derived from food and the energy expended to maintain life and perform physical work. However, quantifying this relationship has proved difficult, in part because the body is composed of multiple components and weight change results from alterations of body composition (i.e., fat versus lean mass). Here, we show that mathematical modeling can provide a general description of how body weight will change over time by tracking the flux balances of the macronutrients fat, protein, and carbohydrates. For a fixed food intake rate and physical activity level, the body weight and composition will approach steady state. However, the steady state can correspond to a unique body weight or a continuum of body weights that are all consistent with the same food intake and energy expenditure rates. Interestingly, existing experimental data on human body weight dynamics cannot distinguish between these two possibilities. We propose experiments that could resolve this issue and use computer simulations to demonstrate how such experiments could be performed.

## Introduction

Obesity, anorexia nervosa, cachexia, and starvation are conditions that have a profound medical, social and economic impact on our lives. For example, the incidence of obesity and its co-morbidities has increased at a rapid rate over the past two decades [1],[2]. These conditions are characterized by changes in body weight (mass) that arise from an imbalance between the energy derived from food and the energy expended to maintain life and perform work. However, the underlying mechanisms of how changes in energy balance lead to changes in body mass and body composition are not well understood. In particular, it is of interest to understand how body composition is apportioned between fat and lean components when the body mass changes and if this energy partitioning can be altered. Such an understanding would be useful for optimizing weight loss treatments in obese subjects to maximize fat loss or weight gain treatments for anorexia nervosa and cachexia patients to maximize lean tissue gain.

To address these issues and improve our understanding of human body weight regulation, mathematical and computational modeling has been attempted many times over the past several decades [3]–[19]. Here we show how models of body composition and mass change can be understood and analyzed within the realm of dynamical systems theory and can be classified according to their geometric structure in the two dimensional phase plane. We begin by considering a general class of macronutrient flux balance equations and progressively introduce assumptions that constrain the model dynamics. We show that two compartment models of fat and lean masses can be categorized into two generic classes. In the first class, there is a unique body composition and mass (i.e. a stable fixed point) that is specified by the diet and energy expenditure. In the second class, there is a continuous curve of fixed points (i.e. an invariant manifold) with an infinite number of possible body compositions and masses at steady state for the same diet and energy expenditure rate. We show that almost all of the models in the literature are in the second class. Surprisingly, the existing data are insufficient to determine which of the two classes pertains to humans. For models with an invariant manifold, we show that an equivalent one dimensional equation for body composition change can be derived. We give numerical examples and discuss possible experimental approaches that may distinguish between the classes.

## Results

### General Model of Macronutrient and Energy Flux Balance

The human body obeys the law of energy conservation [20], which can be expressed as
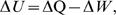
(1)where Δ*U* is the change in stored energy in the body, Δ*Q* is a change in energy input or intake, and Δ*W* is a change in energy output or expenditure. The intake is provided by the energy content of the food consumed. Combustion of dietary macronutrients yields chemical energy and Hess's law states that the energy released is the same regardless of whether the process takes place inside a bomb calorimeter or via the complex process of oxidative phosphorylation in the mitochondria. Thus, the energy released from oxidation of food in the body can be precisely measured in the laboratory. However, there is an important caveat. Not all macronutrients in food are completely absorbed by the body. Furthermore, the dietary protein that is absorbed does not undergo complete combustion in the body, but rather produces urea and ammonia. In accounting for these effects, we refer to the metabolizable energy content of dietary carbohydrate, fat, and protein, which is slightly less than the values obtained by bomb calorimetry. The energy expenditure rate includes the work to maintain basic metabolic function (resting metabolic rate), to digest, absorb and transport the nutrients in food (thermic effect of feeding), to synthesize or break down tissue, and to perform physical activity, together with the heat generated. The energy is stored in the form of fat as well as in lean body tissue such as glycogen and protein. The body need not be in equilibrium for Equation 1 to hold. While we are primarily concerned with adult weight change, Equation 1 is also valid for childhood growth.

In order to express a change of stored energy Δ*U* in terms of body mass *M* we must determine the energy content per unit body mass change, i.e. the energy density ρ*_M_*. We can then set Δ*U* = Δ(ρ*_M_M*). To model the dynamics of body mass change, we divide Equation 1 by some interval of time and take the limit of infinitesimal change to obtain a one dimensional energy flux balance equation:
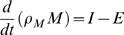
(2)where *I* = *dQ*/*dt* is the rate of metabolizable energy intake and *E* = *dW*/*dt* is the rate of energy expenditure. It is important to note that ρ*_M_* is the energy density of body mass change, which need not be a constant but could be a function of body composition and time. Thus, in order to use Equation 2, the dynamics of ρ*_M_* must also be established.

When the body changes mass, that change will be composed of water, protein, carbohydrates (in the form of glycogen), fat, bone, and trace amounts of micronutrients, all having their own energy densities. Hence, a means of determining the dynamics of ρ*_M_* is to track the dynamics of the components. The extracellular water and bone mineral mass have no metabolizable energy content and change little when body mass changes in adults under normal conditions [21]. The change in intracellular water can be specified by changes in the tissue protein and glycogen. Thus the main components contributing to the dynamics of ρ*_M_* are the macronutrients - protein, carbohydrates, and fat, where we distinguish body fat (e.g. free fatty acids and triglycerides) from adipose tissue, which includes water and protein in addition to triglycerides. We then represent Equation 2 in terms of macronutrient flux balance equations for body fat *F*, glycogen *G*, and protein *P*:
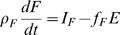
(3)

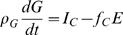
(4)

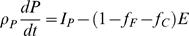
(5)where ρ*_F_* = 39.5 MJ/kg, ρ*_G_* = 17.6 MJ/kg, ρ*_P_* = 19.7 MJ/kg are the energy densities [3], *I_F_*,*I_C_*,*I_P_* are the intake rates, and *f_F_*, *f_C_*, 1−*f_F_*−*f_C_* are the fractions of the energy expenditure rate obtained from the combustion of fat, carbohydrates (glycogen) and protein respectively. The fractions and energy expenditure rate are functions of body composition and intake rates. They can be estimated from indirect calorimetry, which measures the oxygen consumed and carbon dioxide produced by a subject [22]. The intake rates are determined by the macronutrient composition of the consumed food, and the efficiency of the conversion of the food into a utilizable form. Transfer between compartments such as *de novo* lipogenesis where carbohydrates are converted to fat or gluconeogenesis where amino acids are converted into carbohydrates can be accounted for in the forms of *f_F_* and *f_C_*. The sum of Equations 3, 4, and 5 recovers the energy flux balance Equation 2, where the body mass *M* is the sum of the macronutrients *F*, *G*, *P*, with the associated intracellular water, and the inert mass that does not change such as the extracellular water, bones, and minerals, and ρ*_M_* = (ρ*_F_F*+ρ*_G_G*+ρ*_P_P*)/*M*.

The intake and energy expenditure rates are explicit functions of time with fast fluctuations on a time scale of hours to days [23]. However, we are interested in the long-term dynamics over weeks, months and years. Hence, to simplify the equations, we can use the method of averaging to remove the fast motion and derive a system of equations for the slow time dynamics. We do this explicitly in the Methods section and show that the form of the averaged equations to lowest order are identical to Equations 3–5 except that the three components are to be interpreted as the slowly varying part and the intake and energy expenditure rates are moving time averages over a time scale of a day.

The three-compartment flux balance model was used by Hall [3] to numerically simulate data from the classic Minnesota human starvation experiment [21]. In Hall's model, the forms of the energy expenditure and fractions were chosen for physiological considerations. For clamped food intake, the body composition approached a unique steady state. The model also showed that apart from transient changes lasting only a few days, carbohydrate balance is precisely maintained as a result of the limited storage capacity for glycogen. We will exploit this property to reduce the three dimensional system to an approximately equivalent two dimensional system where dynamical systems techniques can be employed to analyze the dynamics.

### Reduced Models

#### Two compartment macronutrient partition model

The three compartment macronutrient flux balance model Equations 3–5 can be reduced to a two dimensional system for fat mass *F* and lean mass *L = M*−*F*, where *M* is the total body mass. The lean mass includes the protein and glycogen with the associated intracellular water along with the mass that does not change appreciably such as the extracellular water and bone. Hence the rate of change in lean mass is given by
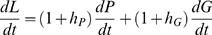
(6)where *h_P_* = 1.6 and *h_G_* = 2.7 are reasonable estimates of the hydration coefficients for the intracellular water associated with the protein and glycogen respectively [3],[24]. (We note that fat is not associated with any water.) The glycogen storage capacity is extremely small compared to the fat and protein compartments. Thus the slow component of glycogen can be considered to be a constant (see Methods). In other words, on time scales much longer than a day, which are of interest for body weight change, we can consider glycogen to be in quasi-equilibrium so that *dG*/*dt* = 0, as observed in numerical simulations [3]. This implies that *f_C_* = *I_C_*/*E*, which can be substituted into Equation 5 to give
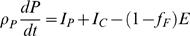
(7)


Substituting Equation 7 and *dG*/*dt* = 0 into Equation 6 leads to the *two compartment macronutrient partition* model
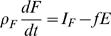
(8)

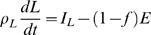
(9)where ρ*_L_* = ρ*_P_*/(1+*h_P_*) = 7.6 MJ/kg, *I_F_* and *I_L_* = *I_P_*+*I_C_* are the intake rates into the fat and lean compartments respectively, *E* = *E*(*I_F_*,*I_L_*,*F*,*L*) is the total energy expenditure rate, and *f* = *f*(*I_F_*,*I_L_*,*F*,*L*) ≡ *f_F_* is the fraction of energy expenditure rate attributed to fat utilization.

We note that *dG*/*dt* = 0 may be violated if the glycogen content is proportional to the protein content, which is plausible because most of the glycogen mass is stored in muscle tissue and may scale with protein mass. We show that this assumption leads to the same two dimensional system. Substituting
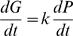
(10)for a proportionality constant *k*, into Equation 4 gives *f_C_E* = *I_C_*−ρ*_C_kdP*/*dt*, which inserted into Equation 5 leads to
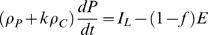
(11)Substituting Equations 10 and 11 into Equation 6 will again result in Equation 9 but with ρ*_L_* = (ρ*_P_*+*k*ρ*_C_*)/((1+*h_P_*+*k*+*kh_G_*). For *k* = 0.044≪1 as suggested by Snyder et al. [25], ρ*_L_* has approximately the same value as before.

Previous studies have considered two dimensional models of body mass change although they were not derived from the three-dimensional macronutrient partition model. Alpert [5]–[7] considered a model with *E* linearized in *F* and *L* and different *f* depending on context. Forbes [8] and Livingston et al. [9] modeled weight loss as a double exponential decay. Although, they did not consider macronutrient flux balance, the dynamics of their models are equivalent to the two dimensional model with *I_F_* and *I_L_* zero, and *E* linear in *F* and *L*.

#### Energy partition model

The two-compartment macronutrient partition model can be further simplified by assuming that trajectories in the *L–F* phase plane follow prescribed paths satisfying
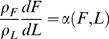
(12)where α(*F*,*L*) is a continuous function [10],[11],[26] that depends on the mechanisms of body weight change. Forbes first hypothesized this stringent constraint after analyzing body composition data collected across a large number of subjects [26],[27]. Forbes postulated that for adults
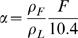
(13)so that
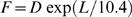
(14)where *D* is a free parameter, and the lean and fat masses are in units of kg. Forbes found that his general relationship (14) was similar whether weight loss is induced by diet or exercise [27]. It is possible that resistance exercise or a significant change in the protein content of the diet may result in a different relationship for α [28]–[30]. Infant growth is an example where α is not well described by the Forbes relationship. Jordan and Hall [11] used longitudinal body composition data in growing infants to determine an appropriate form for α during the first two years of life.

Equation 12 describes a family of *F* vs. *L* curves, parameterized by an integration constant (e.g., *D* in Equation 14). Depending on the initial condition, the body composition moves along one of these curves when out of energy balance. Dividing Equation 8 by Equation 9 and imposing Equation 12 results in
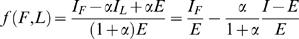
(15)Hall, Bain, and Chow [10] showed that the two compartment macronutrient partition model with Equation 15 using Forbes's law (Equation 13) matched a wide range of data without any adjustable parameters.

Substituting Equation 15 into the macronutrient partition model 8 and 9 leads to the *Energy Partition* model:
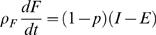
(16)

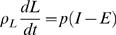
(17)where *p = p*(*F*,*L*) = 1/(1+α) is known as the p-ratio [31]. In the energy partition model, an energy imbalance *I*–*E* is divided between the compartments according to a function *p*(*F*,*L*) that defines the fraction assigned to lean body tissue (mostly protein). Most of the previous models in the literature are different versions of the energy partition model [6], [12]–[18], although none of the authors have noted the connection to macronutrient flux balance or analyzed their models using dynamical systems theory. Some of these previous models are expressed as computational algorithms that can be translated to the form of the energy partition model.

Despite the ubiquity of the energy partition model, the physiological interpretation of the p-ratio remains obscure and is difficult to measure directly. It can be inferred indirectly from *f* (which can be measured by indirect calorimetry) by using Equation 15 [10]. Previous uses of the energy partition model often considered *p* to be a constant [6], [12]–[18], which implies that the partitioning of energy is independent of current body composition and macronutrient composition. This is in contradiction to weight loss data that finds that the fraction of body fat lost does depend on body composition with more fat lost if the body fat is initially higher [26],[32],[33]. However, if α is a weak function of body composition then a constant p-ratio may be a valid approximation for small changes. Flatt [17] considered a model where the p-ratio was constant but included the dynamics for glycogen. His model would be useful when dynamics on short time scales are of interest.

It may sometimes be convenient to express the macronutrient partition model with a unique fixed point as
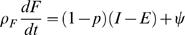
(18)

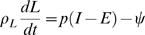
(19)for a function ψ = ψ(*I_F_*,*I_L_*,*F*,*L*), which is zero at the fixed point (*F*_0_,*L*_0_). We use this form in numerical examples below. The fasting model of Song and Thomas [19]) used this form with *I* = 0 and ψ was a function of *F* representing ketone production. Comparing to Equation 8 and Equation 18 gives
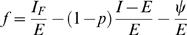
(20)


#### One-dimensional models

The dynamics of the energy partition model Equations 16 and 17 move along fixed trajectories in the *L*–*F* plane. Thus a further simplification to a one dimensional model is possible by finding a functional relationship between *F* and *L* so that one variable can be eliminated in favor of the other. Such a function exists if Equation 12 has a unique solution, which is guaranteed in some interval of *L* if α(*F*,*L*) and ∂α/∂*F* are continuous functions of *F* and *L* on a rectangle containing this interval. These are sufficient but not necessary conditions.

Suppose a relationship *F* = φ(*L*) can be found between *F* and *L*. Substituting this relationship into Equation 16 and Equation 17 and adding the two resulting equations yields the one dimensional equation
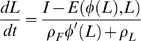
(21)We can obtain a dynamical equation for body mass by expressing the body mass as *M = L+*φ*(L)*. If we can invert this relationship uniquely and obtain *L* as a function of *M*, then this can be substituted into Equation 21 to obtain a dynamical equation for *M*.

As an example, assume *p* to be a constant, which was used in [6], [12]–[18]. This implies that the phase orbits are a family of straight lines of the form *F* = β*L+C*≡ φ(*L*) where β = ρ*_L_*(1−*p*)/(ρ*_F_p*) and *C* is a constant that is specified by the initial body composition. This results in
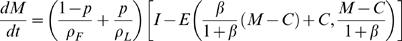
(22)Linearizing Equation 22 around a mass *M*_0_ gives
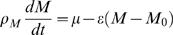
(23)where ρ*_M_* = ρ*_F_*ρ*_L_*/(ρ*_L_*+(ρ*_F_*−ρ*_L_*)*p*), μ = *I*−*E*(*F*(*M*_0_),*L*(*M*_0_)), and 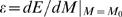. This is the form used in [18].

If Equations 16 and 17 are constrained to obey the phase plane paths of Forbes's law, then a reduction to a one dimensional equation can also be made. Using Equation 14 (i.e., φ(*L*) = *D*exp(*L*/10.4)) in Equation 21 yields
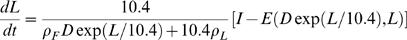
(24)Similarly, a one dimensional equation for the fat mass has the form
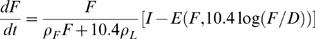
(25)Since the mass functions *M = L*+*D*exp(*L*/10.4) or *M = F*+10.4log(*F*/*D*) cannot be inverted in closed form, an explicit one dimensional differential equation in terms of the mass cannot be derived. However, the dynamics of the mass is easily obtained using either Equation 24 or Equation 25 together with the relevant mass function. For large changes in body composition, the dynamics could differ significantly from the constant *p* models 22 or 23.

The one dimensional model gives the dynamics of the energy partition model along a fixed trajectory in the *F*–*L* plane. The initial body composition specifies the constant *C* or *D* in the above equations. A one dimensional model will represent the energy partition model even if the intake rate is time dependent. Only for a perturbation that directly alters body composition will the one dimensional model no longer apply. However, after the perturbation ceases, the one dimensional model with a new constant will apply again.

### Existence and Stability of Body Weight Fixed Points

The various flux balance models can be analyzed using the methods of dynamical systems theory, which aims to understand dynamics in terms of the geometric structure of possible trajectories (time courses of the body components). If the models are smooth and continuous then the global dynamics can be inferred from the local dynamics of the model near fixed points (i.e. where the time derivatives of the variables are zero). To simplify the analysis, we consider the intake rates to be clamped to constant values or set to predetermined functions of time. We do not consider the control and variation of food intake rate that may arise due to feedback from the body composition or from exogenous influences. We focus only on what happens to the food once it is ingested, which is a problem independent of the control of intake. We also assume that the averaged energy expenditure rate does not depend on time explicitly. Hence, we do not account for the effects of development, aging or gradual changes in lifestyle, which could lead to an explicit slow time dependence of energy expenditure rate. Thus, our ensuing analysis is mainly applicable to understanding the slow dynamics of body mass and composition for clamped food intake and physical activity over a time course of months to a few years.

Dynamics in two dimensions are particularly simple to analyze and can be easily visualized geometrically [34],[35]. The one dimensional models are a subclass of two dimensional dynamics. Three dimensional dynamical systems are generally more difficult to analyze but Hall [3] found in simulations that the glycogen levels varied over a small interval and averaged to an approximate constant for time periods longer than a few days, implying that the slow dynamics could be effectively captured by a two dimensional model. Reduction to fewer dimensions is an oft-used strategy in dynamical systems theory. Hence, we focus our analysis on two dimensional dynamics.

In two dimensions, changes of body composition and mass are represented by trajectories in the *L–F* phase plane. For *I_F_* and *I_L_* constant, the flux balance model is a two dimensional autonomous system of ordinary differential equations and trajectories will flow to attractors. The only possible attractors are infinity, stable fixed points or stable limit cycles [34],[35]. We note that fixed points within the context of the model correspond to states of flux balance. The two compartment macronutrient partition model is completely general in that all possible autonomous dynamics in the two dimensional phase plane are realizable. Any two or one dimensional autonomous model of body composition change can be expressed in terms of the two dimensional macronutrient partition model.

**Figure 1:**
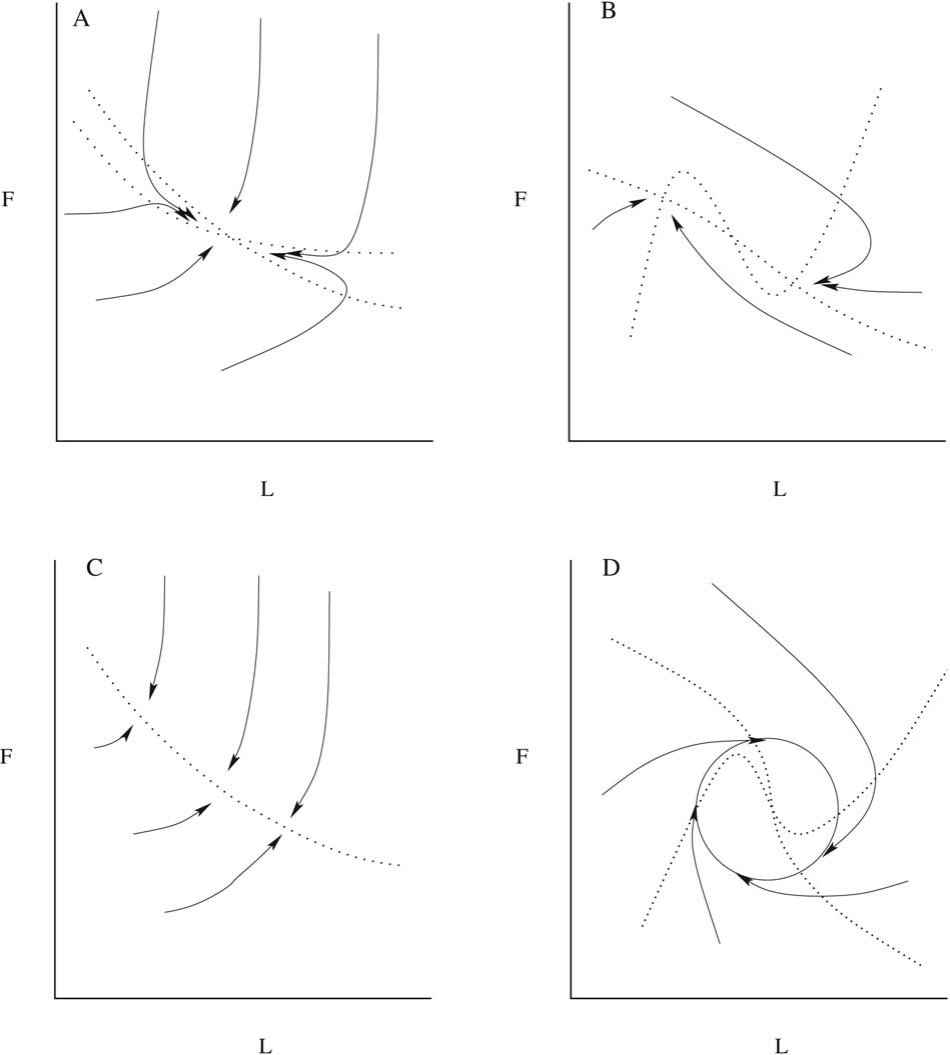
Possible trajectories (solid lines) for different initial conditions and nullclines (dotted lines) in the *L–F* phase plane for models with a stable fixed point (A), multi-stability with two stable fixed points separated by one unstable saddle point (B), an attracting invariant manifold (C), and a limit cycle attractor (D).

Physical viability constrains *L* and *F* to be positive and finite. For differentiable *f* and *E*, the possible trajectories for fixed intake rates are completely specified by the dynamics near fixed points of the system. Geometrically, the fixed points are given by the intersections of the nullclines in the *L–F* plane, which are given by the solutions of *I_F_*−*fE* = 0 and *I_L_* = (1−*f*)*E* = 0. Example nullclines and phase plane portraits of the macronutrient model are shown in Figure 1. If the nullclines intersect once then there will be a single fixed point and if it is stable then the steady state body composition and mass are uniquely determined. Multiple intersections can yield multiple stable fixed points implying that body composition is not unique [4]. If the nullclines are collinear then there can be an attracting one dimensional invariant manifold (continuous curve of fixed points) in the *L*–*F* plane. In this case, there are an infinite number of possible body compositions for a fixed diet. As we will show, the energy partition model implicitly assumes an invariant manifold. If a single fixed point exists but is unstable then a stable limit cycle may exist around it.

The fixed point conditions of Equations 8 and 9 can be expressed in terms of the solutions of
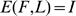
(26)

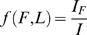
(27)where *I* = *I_F_*+*I_L_*, and we have suppressed the functional dependence on intake rates. These fixed point conditions correspond to a state of flux balance of the lean and fat components. Equation 26 indicates a state of energy balance while Equation 27 indicates that the fraction of fat utilized must equal the fraction of fat in the diet. Stability of a fixed point is determined by the dynamics of small perturbations of body composition away from the fixed point. If the perturbed body composition returns to the original fixed point then the fixed point is deemed stable. We give the stability conditions in Methods.

The functional dependence of *E* and *f* on *F* and *L* determine the existence and stability of fixed points. As shown in Methods, an isolated stable fixed point is guaranteed if *f* is a monotonic increasing function of *F* and a monotonic decreasing function of *L*. If one of the fixed point conditions automatically satisfies the other, then instead of a fixed point there will be a continuous curve of fixed points or an invariant manifold. For example, if the energy balance condition 26 automatically satisfies the fat fraction condition 27, then there is an invariant manifold defined by *I = E(F,L)*. The energy partition model has this property and thus has an invariant manifold rather than an isolated fixed point. This can be seen by observing that for *f* given by Equation 15, Equation 26 automatically satisfies condition 27. An attracting invariant manifold implies that the body can exist at any of the infinite number of body compositions specified by the curve *I = E*(*F*,*L*) for clamped intake and energy expenditure rates (see Figure 1C). Each of these infinite possible body compositions will result in a different body mass *M = F*+*L* (except for the unlikely case that *E* is a function of the sum *F*+*L*). The body composition is marginally stable along the direction of the invariant manifold. This means that in flux balance, the body composition will remain at rest at any point on the invariant manifold. A transient perturbation along the invariant manifold will simply cause the body composition to move to a new position on the invariant manifold. The one dimensional models have a stable fixed point if the invariant manifold is attracting. We also show in Methods that for multiple stable fixed points or a limit cycle to exist, *f* must be nonmonotonic in *L* and be finely tuned. The required fine-tuning makes these latter two possibilities much less plausible than a single fixed point or an invariant manifold.

Data suggest that *E* is a monotonically increasing function of *F* and *L* [36]. The dependence of *f* on *F* and *L* is not well established and the form of *f* depends on multiple interrelated factors. In general, the sensitivity of various tissues to the changing hormonal milieu will have an overall effect on both the supply of macronutrients as well as the substrate preferences of various metabolically active tissues. On the supply side, we know that free fatty acids derived from adipose tissue lipolysis increase with increasing body fat mass which thereby increase the daily fat oxidation fraction, *f*, as *F* increases [37]. Furthermore, reduction of *F* with weight loss has been demonstrated to decrease *f* [38]. Similarly, whole-body proteolysis and protein oxidation increases with lean body mass [39],[40] implying that *f* should be a decreasing function of *L*. In further support of this relationship, body builders with significantly increased *L* have a decreased daily fat oxidation fraction versus control subjects with similar *F* [41]. Thus a stable isolated fixed point is consistent with this set of data.

### Implications for Body Mass and Composition Change

We have shown that all two dimensional autonomous models of body composition change generically fall into two classes - those with fixed points and those with invariant manifolds. In the case of a stable fixed point, any temporary perturbation of body weight or composition will be corrected over time (i.e., for all things equal, the body will return to its original state). An invariant manifold allows the possibility that a transient perturbation could lead to a permanent change of body composition and mass.

**Figure 2:**
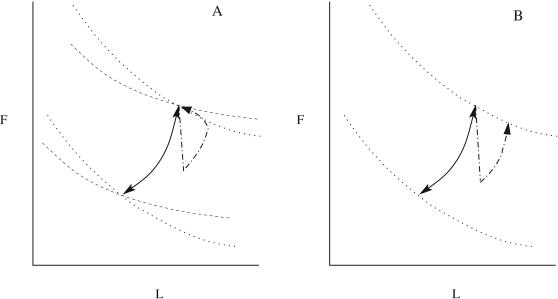
An example of a situation where the intake or energy expenditure rate is changed from one clamped value to another and then returned. (A) Fixed point case. (B) Invariant manifold case. Dotted lines represent nullclines. In both cases, the body composition follows a fixed trajectory and returns to the original steady state (solid curves). However, if the body composition is perturbed directly (dashed-dot curves) then the body composition will flow to same point in (A) but to a different point in (B).

At first glance, these differing properties would appear to point to a simple way of distinguishing between the two classes. However, the traditional means of inducing weight change namely diet or altering energy expenditure through aerobic exercise, turn out to be incapable of revealing the distinction. For an invariant manifold, any change of intake or expenditure rate will only elicit movement along one of the prescribed *F* vs. *L* trajectories obeying Equation 12, an example being Forbes's law (14). As shown in Figure 2, a change of intake or energy expenditure rate will change the position of the invariant manifold. The body composition that is initially at one point on the invariant manifold will then flow to a new point on the perturbed invariant manifold along the trajectory prescribed by (12). If the intake rate or energy expenditure is then restored to the original value then the body composition will return along the same trajectory to the original steady state just as it would in a fixed point model (see Figure 2 solid curves). Only a perturbation that moves the body composition off of the fixed trajectory could distinguish between the two classes. In the fixed point case (Figure 2A dashed-dot curve), the body composition would go to the same steady state following the perturbation to body composition but for the invariant manifold case (Figure 2B dashed-dot curve), it would go to another steady state.

Perturbations that move the body composition off the fixed trajectory can be done by altering body composition directly or by altering the fat utilization fraction *f*. For example, body composition could be altered directly through liposuction and *f* could be altered by administering compounds such as growth hormone. Resistance exercise may cause an increase in lean muscle tissue at the expense of fat. Exogenous hormones, compounds, or infectious agents that change the propensity for fat versus carbohydrate oxidation (for example, by increasing adipocyte proliferation and acting as a sink for fat that is not available for oxidation [42]–[44]), would also perturb the body composition off of a fixed *F* vs. *L* curve by altering *f*. If the body composition returned to its original state after such a perturbation then there is a unique fixed point. If it does not then there could be an invariant manifold although multiple fixed points are also possible.

We found an example of one clinical study that bears on the question of whether humans have a fixed point or an invariant manifold. Biller et al. investigated changes of body composition pre- and post-growth hormone therapy in forty male subjects with growth hormone deficiency [45]. Despite significant changes of body composition induced by 18 months of growth hormone administration, the subjects returned very closely to their original body composition 18 months following the removal of therapy. However, there was a slight (2%) but significant increase in their lean body mass compared with the original value. Perhaps not enough time had elapsed for the lean mass to return to the original level. Alternatively, the increased lean mass may possibly have been the result of increased bone mineral mass and extracellular fluid expansion, both of which are known effects of growth hormone, but were assumed to be constant in the body composition models. Therefore, this clinical study provides some evidence in support of a fixed point, but it has not been repeated and the result was not conclusive. Using data from the Minnesota experiment [21] and the underlying physiology, Hall [3] proposed a form for *f* that predicts a fixed point. On the other hand, Hall, Bain, and Chow [10] showed that an invariant manifold model is consistent with existing data of longitudinal weight change but these experiments only altered weight through changes in caloric intake so this cannot rule out the possibility of a fixed point. Thus it appears that existing data is insufficient to decide the issue.

### Numerical Simulations

We now consider some numerical examples using the macronutrient partition model in the form given by Equations 18 and 19, with a p-ratio consistent with Forbes's law (13) (i.e. *p* = 2/(2+*F*), where *F* is in units of kg). Consider two cases of the model. If ψ = 0 then the model has an invariant manifold and body composition moves along a fixed trajectory in the *L*–*F* plane. If ψ is nonzero, then there can be an isolated fixed point. We will show an example where if the intake energy is perturbed, the approach of the body composition to the steady state will be identical for both cases but if body composition is perturbed, the body will arrive at different steady states.

For every model with an invariant manifold, a model with a fixed point can be found such that trajectories in the *L–F* plane resulting from energy intake perturbations will be identical. All that is required is that ψ in the fixed point model is chosen such that the solution of ψ (*F,L*) = 0 defines the fixed trajectory of the invariant manifold model. Using Forbes's law (14), we choose ψ = 0.05(*F*−0.4 exp(*L*/10.4))/*F*. We then take a plausible energy expenditure rate of *E* = 0.14*L*+0.05*F*+1.55, where energy rate has units of MJ/day and mass has units of kg. This expression is based on combining cross-sectional data [36] for resting energy with a contribution of physical activity of a fairly sedentary person [3]. Previous models propose similar forms for the energy expenditure [5],[7],[13],[18].

**Figure 3:**
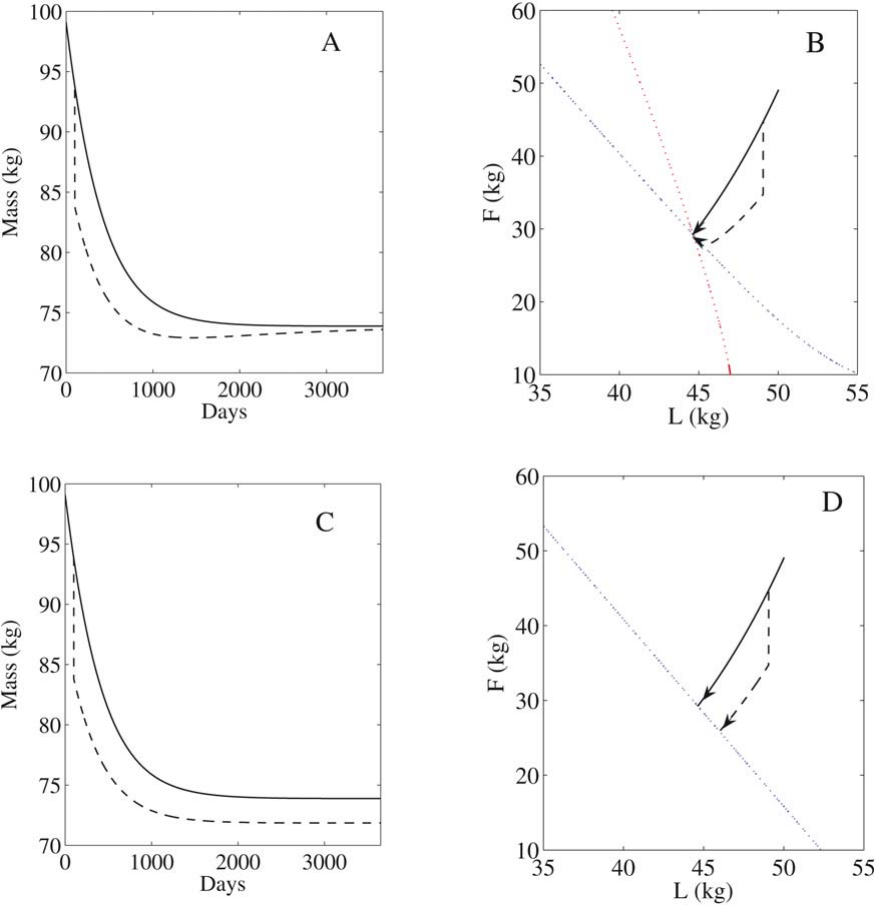
Time dependence of body mass and *F* vs. *L* trajectories. In all the Figures, the solid line is for an intake reduction from 12 MJ/day to 10 J/day and the dashed line is for the same reduction but with a removal of 10 kg of fat at day 100. Time dependence of body mass for the fixed point model (A). Trajectories in the *F* vs. *L* phase plane for the fixed point model (B). Dotted lines are the nullclines. Time dependence (C) and phase plane (D) of the invariant manifold model for the same conditions.

Figure 3 shows the time dependence of body mass and the *F* vs. *L* trajectories of the two model examples given a reduction in energy intake rate from 12 MJ/day to 10 MJ/day starting at the same initial condition. The time courses are identical for body composition and mass. The mass first decreases linearly in time but then saturates to a new stable fixed point. The dashed line represents the same intake rate reduction but with 10 kg of fat removed at day 100. For the invariant manifold model, the fat perturbation permanently alters the final body composition and body mass, whereas in the fixed point model it only has a transient effect. In the fixed point model, the body composition can ultimately exist only at one point given by the intersection of the nullclines (i.e., solution of *I = E* and ψ = 0). For the invariant manifold, the body composition can exist at any point on the *I* = *E* curve (dotted line in Figure 2D). Since a ψ can always be found so that a fixed point model and an invariant manifold model have identical time courses for body composition and mass, a perturbation in energy intake can never discriminate between the two possibilities.

The time constant to reach the new fixed point in the numerical simulations is very long. This slow approach to steady state (on the order of several years for humans) has been pointed out many times previously [3],[5],[7],[13],[18]. A long time constant will make experiments to distinguish between a fixed point and an invariant manifold difficult to conduct. Experimentally reproducing this example would be demanding but if the time variation of the intake rates and physical activity levels were small compared to the induced change then the same result should arise qualitatively. Additionally, the time constant depends on the form of the energy expenditure. There is evidence that the dependence of energy expenditure on *F* and *L* for an individual is steeper than for the population due to an effect called adaptive thermogenesis [46], thus making the time constant shorter.

## Discussion

In this paper we have shown that all possible two dimensional autonomous models for lean and fat mass are variants of the macronutrient partition model. The models can be divided into two general classes - models with isolated fixed points (most likely a single stable fixed point) and models with an invariant manifold. There is the possibility of more exotic behavior such as multi-stability and limit cycles but these require fine-tuning and thus are less plausible. Surprisingly, experimentally determining if the body exhibits a fixed point or an invariant manifold is nontrivial. Only perturbations of the body composition itself apart from dietary or energy expenditure interventions or alterations of the fraction of energy utilized as fat can discriminate between the two possibilities. The distinction between the classes is not merely an academic concern since this has direct clinical implications for potential permanence of transient changes of body composition via such procedures as liposuction or temporary administration of therapeutic compounds.

Our analysis considers the slow dynamics of the body mass and composition where the fast time dependent hourly or daily fluctuations are averaged out for a clamped average food intake rate. We also do not consider a slow explicit time dependence of the energy expenditure. Such time dependence could arise during development, aging or gradual changes in lifestyle where activity levels differ. Thus our analysis is best suited to modeling changes over time scales of months to a few years in adults. We do not consider any feedback of body composition on food intake, which is an extremely important topic but beyond the scope of this paper.

Previous efforts to model body weight change have predominantly used energy partition models that implicitly contain an invariant manifold and thus body composition and mass are not fully specified by the diet. If the body does have an invariant manifold then this fact puts a very strong constraint on the fat utilization fraction *f*. Hall [3] considered the effects of carbohydrate intake on lipolysis and other physiological factors to conjecture a form of *f* that does not lead to an invariant manifold. However, our analysis and numerical examples show that the body composition could have an invariant manifold but behave indistinguishably from having a fixed point. Also, the decay to the fixed point could take a very long time, possibly as long as a decade giving the appearance of an invariant manifold. Only experiments that perturb the fat or lean compartments independently can tell.

## Methods

### Method of Averaging

The three compartment macronutrient flux balance Equations 3–5 are a system of nonautonomous differential equations since the energy intake and expenditure are explicitly time dependent. Food is ingested over discrete time intervals and physical activity will vary greatly within a day. However, this fast time dependence can be viewed as oscillations or fluctuations on top of a slowly varying background. It is this slower time dependence that governs long-term body mass and composition changes that we are interested in. For example, if an individual had the exact same schedule with the same energy intake and expenditure each day, then averaged over a day, the body composition would be constant. If the daily averaged intake and expenditure were to gradually change on longer time scales of say weeks or months then there would be a corresponding change in the body composition and mass. Given that we are only interested in these slower changes, we remove the short time scale fluctuations by using the method of averaging to produce an autonomous system of *averaged* equations valid on longer time scales.

We do so by introducing a second “fast” time variable τ = *t*/ε, where ε is a small parameter that is associated with the slow changes in body composition and let all time dependent quantities be a function of both *t* and τ. For example, if *t* is measured in units of days and τ is measured in units of hours then ε∼1/24. Inserting into Equations 3–5 and using the chain rule yields
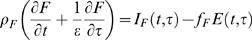
(28)

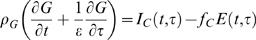
(29)

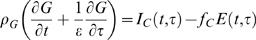
(29)We then consider the three body compartments to have expansions of the form
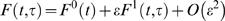
(31)

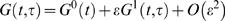
(32)

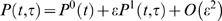
(33)where 〈*F* ^1^〉 = 〈*P* ^1^〉 = 〈*G* ^1^〉 = 0 for a time average defined by 
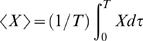
 and *T* represents an averaging time scale of a day. The fast time dependence can be either periodic or stochastic. The important thing is that the time average over the fast quantities is of order ε or higher. We then expand the energy expenditure rate and expenditure fractions to first order in ε:
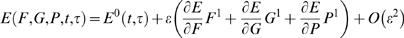
(34)

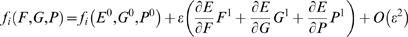
(35)where *E* ^0^(*t*,τ)≡*E*(*F* ^0^,*G* ^0^,*P* ^0^,*t*,τ)+*O*(ε^2^) and *i*∈{*F*,*G,P*}. We assume that the expenditure fractions depend on time only through the body compartments. Substituting these expansions into Equations 28–30 and taking lowest order in ε gives
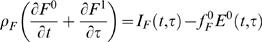
(36)

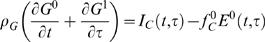
(37)

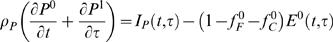
(38)


Taking the moving time average of Equations 36–38 and requiring that 〈∂*F* ^1^/∂τ〉, 〈∂*G* ^1^/∂τ〉, and 〈∂*P* ^1^/∂τ〉 are of order ε or higher leads to the averaged equations:
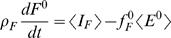
(39)

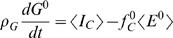
(40)

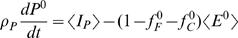
(41)In the main text we only consider the slow time scale dynamics so we drop the superscript and bracket notation for simplicity. Hence, the system (3–5) can be thought of as representing the lowest order time averaged macronutrient flux balance equations. We note that in addition to the daily fluctuations of meals and physical activity, there can also be fluctuations in food intake from day to day [23]. Our averaging scheme can be used to average over these fluctuations as well by extending the averaging time *T*. A difference in the choice of *T* will only result in a different interpretation of the averaged quantities.

### Stability Conditions for Fixed Points

The dynamics near a fixed point (*F*_0_,*L*_0_) are determined by expanding *fE* and (1−*f*)*E* to linear order in δ*F* = *F*−*F*_0_ and δ*L* = *L*−*L*_0_ [34],[35]. Assuming solutions of the form exp(λ*t*) yields an eigenvalue problem with two eigenvalues given by 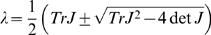 where
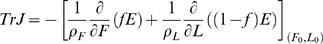
(42)and
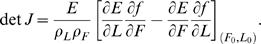
(43)A fixed point is stable if and only if Tr *J*<0 and det *J*>0. In the case of an invariant manifold, det*J* = 0, so the eigenvalues are Tr *J* and 0. The zero eigenvalue reflects the marginal stability along the invariant manifold, which is an attractor if Tr *J*<0. An attracting invariant manifold implies a stable fixed point in the corresponding one dimensional model. Unstable fixed points are either unstable nodes, saddle points or unstable spirals. In the case of unstable spirals, a possibility is a limit cycle surrounding the spiral arising from a Hopf bifurcation, where Tr *J* = 0 and det *J*>0. In this case, body composition and mass would oscillate even if the intake rates were held constant. The frequency and amplitude of the oscillations may be estimated near a supercritical Hopf bifurcation by transforming the equations to normal form. Stability of a fixed point puts constraints on the form of *f*. Physiological considerations and data imply that ∂*E*/∂*L*>∂*E*/∂*F*>0 [3],[36]. Thus we can set ∂*E*/∂*F* = δ∂*E*/∂*L* where δ <1 (the derivatives are evaluated at the fixed point). Then det*J*>0 implies that
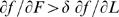
(44)and Tr *J*<0 implies
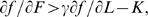
(45)where *K* = [δ*f*+γ (1−*f*)](∂*E*/∂*L*)/*E*>0 and γ = ρ*_F_*/ρ*_L_*≈5.2. Hence ∂*f*/∂*F*>0 and ∂*f*/∂*L*<0 guarantees stability of a fixed point. In other words, if *f* increases monotonically with *F* and decreases monotonically with *L* then there will be a unique stable fixed point. For an invariant manifold, *f* is given by Equation 15, which immediately satisfies det*J* = 0; Tr*J*<0 is guaranteed if *E* is monotonically increasing in *F* and *L*. For a Hopf bifurcation, we require ∂*f*/∂*F* = γ∂*f*/∂*L*−*K* and Equation 44, implying (γ−δ)∂*f*/∂*L*−*K*>0. Since γ>δ, *f* must increase with *L* for the possibility of a limit cycle. However, to ensure that trajectories remain bounded *f* must decrease with *L* for very small and large values of *L*. Hence, *f* must be nonmonotonic in *L* for a limit cycle to exist. This can also be seen from an application of Bendixson's criterion [35], which states that a limit cycle cannot exist in a given region of the *L*–*F* plane if
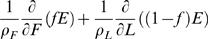
(46)does not change sign in that region. In addition, the other parameters must be fine tuned for a limit cycle (see Figure 1D). Similarly, as seen in Figure 1C), for multi-stability to exist, nonmonotonicity and fine tuning are also required.
